# Evaluating Tinnitus in Children: A Comprehensive Review of Diagnostic Methods and Treatment Strategies

**DOI:** 10.7759/cureus.105159

**Published:** 2026-03-13

**Authors:** Sarika Palve, Yuwaraj D Kale, Dipti Nene, Purti Dilip Barhate, Snehal Vitthalrao Garhate, Vinodini Vijay Payghan, Ashvini Ashokrao Parkhi

**Affiliations:** 1 Department of Shalakyatantra, Bhausaheb Mulak Ayurved College and Research Hospital, Butibori, IND; 2 Department of Shalakyatantra, Maharashtra University of Health Sciences, Nashik, IND; 3 Department of Shalakyatantra, B. R. Harne Ayurvedic Medical College, Vangani, IND; 4 Department of Balrog, Bharati Vidyapeeth (Deemed to be University), Pune, IND; 5 Department of Roga Nidan Evum Vikriti Vigyan, Bhausaheb Mulak Ayurved College and Research Hospital, Butibori, IND; 6 Department of Roga Nidan Evum Vikriti Vigyan, Maharashtra University of Health Sciences, Nashik, IND

**Keywords:** audiological assessment, children, diagnostic strategies, pediatric tinnitus, treatment approaches

## Abstract

Tinnitus in children represents a clinically significant condition with potential influence on auditory maturation, emotional stability, concentration, sleep quality, and academic performance. Limited symptom expression, developmental variability, and diverse underlying etiologies often delay recognition, creating the need for clearer diagnostic pathways. The objective of this narrative review is to consolidate contemporary understanding of diagnostic methods and treatment strategies applicable to pediatric tinnitus, with emphasis on developmental and neurocognitive factors that shape symptom perception and functional impact. A narrative methodology was applied, drawing on peer-reviewed publications from 2015 to 2025 identified across major scientific databases, with inclusion of studies providing clinically relevant pediatric information. The review outlines diagnostic approaches involving behavioral audiometry, objective auditory measures, medical and otologic evaluation, psychological assessment, and emerging digital tools, each supporting refined interpretation of peripheral and central auditory processes. Treatment strategies encompass sound-based interventions, amplification for coexisting hearing deficits, tinnitus retraining frameworks, cognitive-behavioral methods, mindfulness practices, and supportive family-centered engagement. The synthesis highlights the importance of multidisciplinary involvement and structured long-term care to accommodate developmental transitions. The conclusion emphasizes the need for standardized diagnostic protocols, validated objective tools, and expanded longitudinal evidence to enhance accuracy and guide targeted, developmentally appropriate management for children with tinnitus.

## Introduction and background

Pediatric tinnitus is a clinically significant but often under-researched condition of the auditory system that is typified by the perception of sound when not caused by an external source [1]. The effect occurs within a broad range of developmental stages, such as both children with normal hearing thresholds and children with already existing auditory impairment [2]. Reported prevalence has been seen to be 7-30% based on the differences in assessment tools, characteristics of the cohort, and the weaknesses in the methodology [3]. The emergent auditory system has a higher neural plasticity that manifests symptom patterns and influences cortical responses that are not similar to those of adults. These developmental traits render presentation clinically unpredictable and hard to evaluate in a standardized way, particularly in children at an earlier stage of their development, as they do not have well-established language or metacognitive abilities to characterize inner-ear experiences [4]. Children are normally diagnosed late with tinnitus due to difficulties in perceptual experience description, poor symptom awareness, and a similar display of behavioral symptoms [5]. The symptoms may be misinterpreted as inattention or emotional withdrawal, which can lead clinicians to focus primarily on evaluating the auditory system rather than recognizing the underlying cause [1]. This misperception is especially alarming when the neurocognitive and language development and emotional stability are at critical stages of development, as chronic tinnitus may disrupt focus, education, speech and language growth, and emotional stability [6]. The co-occurrence of co-existing conditions, such as otitis media, threshold shifts due to noise, congenital anomalies, vestibular dysfunction, head trauma, exposure to ototoxics, and systematic assessment of both symptomatic and high-risk groups, enhances the risk of tinnitus even further [3].

The assessment strategies that are applied in adults are often modified to be used in pediatrics, but there is not much validation between the stages of development. The tools that involve behavioral audiometry, psychoacoustic matching, and questionnaire-based tools also need modifications related to age, especially among children with limited attention span or communication disabilities [7]. Objective measures such as otoacoustic emission, auditory brainstem response, middle ear muscle reflex testing, and electrophysiological measures give supplementary data to behavioral results [8]. A large number of these tools are standardized to adequately assess tinnitus in children, and a part of the reason why there is a difference in clinical practice across institutions. Heterogeneous implementation of tested age-specific measures impairs the diagnostic reliability and makes cross-study comparison difficult [4].

Therapeutic approaches to pediatric tinnitus have continued to increase in a slow manner, albeit unevenly, in support of evidence. Sound-based intervention, such as the use of hearing aids optimally in instances of hearing loss and organized sound enrichment, proves helpful in alleviating the severity of symptoms and eliciting habituation [6]. The cognitive-behavioral techniques offer more organized coping mechanisms, lessen distress, and enhance sleep patterns, with the available evidence being mostly based on small cohorts or extrapolated on older populations [1]. Both mindfulness-based and relaxation techniques seem to be potentially effective in improving emotional control and alleviating tension and distress associated with tinnitus in older children [1]. Pharmacological therapy is viewed with reservations because of neurobiological developmental issues and minimal reliable evidence of effectiveness [9]. Multidisciplinary and family-centered models of treatment deserve the broader psychological effects, such as irritability, diminished academic activity, sleeping difficulties, and emotional dysregulation [10]. Family involvement has been linked to better adherence, less distress, and environmental modification that encourages coping [7]. Standardized treatment guidelines in pediatrics are not yet available, and there is limited long-term outcome data to support the knowledge of the symptom patterns and responsiveness to treatment into adolescence and early adulthood [4].

Objectives of the review

The review aims to synthesize contemporary diagnostic and therapeutic strategies relevant to tinnitus in children, emphasizing developmental considerations that influence assessment accuracy and management outcomes. It further seeks to highlight existing evidence gaps to support the advancement of standardized and developmentally appropriate clinical pathways.

Methodology

Targeted searches in PubMed, Scopus, Web of Science, and Google Scholar provided this extensive review based on a desire to find publications on the topic of tinnitus among children published in English between 2015 and 2025. The keywords were combinations of “pediatric tinnitus”, “tinnitus in children”, “diagnostic assessment”, “audiological testing”, and “treatment”, and other keywords were “otoacoustic emissions”, “auditory brainstem response”, “sound therapy”, “hearing aids”, “cognitive behavioural therapy”, “mindfulness”, “relaxation therapy”, and “pharmacological treatment”. Manual screening of reference lists of the relevant studies was also performed in order to identify other sources.

The inclusion criterion was that the studies had to discuss tinnitus in children and provide any clinically significant information about the nature of the symptoms, methods of assessment, audiological evaluation, or treatment. Studies that dealt solely with adult tinnitus, non-clinical commentary that was not related to pediatric practice, and non-peer-reviewed articles were excluded. The literature included was synthesized to determine the major evidence on the patterns of diagnostics and treatment of pediatric tinnitus. The final synthesis incorporated 48 peer-reviewed studies published between 2015 and 2025.

## Review

Epidemiology and clinical presentation of pediatric tinnitus

Population-based studies show that there is a great variation in the reported prevalence of pediatric tinnitus, which is a measure of heterogeneity in study design, symptom inquiry techniques, and age stratification techniques [11]. The clinical picture among children is often heterogeneous; the symptoms have different persistence, perceived laterality, and functional effects, and these aspects are commonly dependent on the developmental stage and conditions co-presenting with deafness [12]. They are more often and have more clinically significant symptom profiles in response to cohorts that have entered into otologic disorders, noise exposure, congenital hearing impairment, and middle ear pathology [13]. Additional risk factors have also been reported in correlation with chronic otitis media, vestibular dysfunction, head or neck trauma, and ototoxic agents [10]. There is some emerging evidence that genetics and neurodevelopmental aspects can be a cause of variable symptoms, where some children are more sensitive to auditory stimuli or have differences in central auditory processing, which can adjust tinnitus perception [14,15].

Pathophysiological mechanisms relevant to children

The pathogenesis of tinnitus in children involves a complex interaction between peripheral auditory system dysfunction and neuroplastic changes occurring during active stages of neural development [16]. The peripheral contributors usually start in the cochlea, and they can be the outer hair cells, inner hair cells, stria vascularis, and the spiral ganglion neurons [17]. Disruption of these structures disrupts mechanoelectrical transduction or impairs the work of cochlear amplifiers, resulting in abnormal neural signals transmitted to central auditory pathways [14]. These changes can be after middle ear inflammation, birth variation, acoustic trauma, or metabolic imbalance on cochlear functioning. The central auditory circuits have a significant influence on the maintenance of tinnitus when deviant signals are referred to the higher centers [18]. Amplified spontaneous neural activity, amplified neural synchrony, and central gain in the auditory nuclei produce a high level of internal activity that is perceived as sound [19]. The tonotopic reorganization of auditory cortical areas could occur due to a sustained, inconsistent, or low sensory input, and this could lead to changes in the frequency representation that reinforce the phantom percepts [19].

Neuroplasticity shows high effects in childhood because of the increased responsiveness of the developing neural circuits. High plastic potential could enhance compensatory cortical changes following peripheral disruption, modify excitatory/inhibitory interactions, or strengthen maladaptive circuits associated with tinnitus production [12]. The contribution of disturbance in thalamocortical communication to the continuation of symptoms also exists, especially among children with episodic conductive impairment or childhood hearing loss. Limbic pathways are in progressive development, and higher interconnection between the limbic and auditory networks can bring a higher level of salience to tinnitus, which adds to sleep disturbance, poor concentration, or higher distress [20]. Table 1 shows the principal mechanisms, anatomical sites, functional disturbances, neural outcomes, and related clinical manifestations associated with tinnitus in children.

**Table 1:** Pathophysiological contributors to tinnitus in the pediatric auditory system Compiled by the authors based on information synthesized from [11,14,19,21,22,23].

Mechanism	Primary Site Involved	Functional Disturbance	Typical Neural Outcome	Potential Clinical Manifestation	References
Cochlear alteration	Outer hair cells, inner hair cells, stria vascularis	Distorted mechanoelectrical transduction	Irregular auditory nerve signalling	Continuous tonal or noise-like perception	[21]
Spiral ganglion involvement	Primary auditory neurons	Impaired signal encoding	Intermittent neural discharge	Fluctuating internal sound	[14]
Central gain enhancement	Brainstem and midbrain nuclei	Increased spontaneous neural activity	Amplified auditory pathway signalling	Heightened sound awareness or hypersensitivity	[11]
Cortical reorganization	Primary auditory cortex	Altered tonotopic mapping	Expanded or shifted frequency representation	Persistent phantom percept	[19]
Limbic–auditory interaction	Amygdala–auditory cortex networks	Elevated emotional salience attribution	Reinforced perceptual attention and distress signalling	Sleep disturbance, reduced focus, emotional reactivity	[22]
Somatosensory modulation (e.g., bruxism, temporomandibular dysfunction)	Trigeminal nerve pathways, temporomandibular joint, dorsal cochlear nucleus	Aberrant somatosensory input influencing auditory nuclei	Altered cross-modal neural firing within brainstem auditory circuits	Movement- or jaw-position–modulated tinnitus; fluctuation with clenching or mastication	[23]

Diagnostic challenges in pediatric tinnitus assessment

The evaluation of tinnitus among children is highly complex because of developmental, behavioral, and clinical issues that affect recognition of symptoms and the accuracy of diagnosis [23]. Verbal description of auditory impressions of the inner ear is usually weak in early life, which makes it impossible to separate tinnitus and overall discomfort or non-specific behavioral reactions [17]. Poor abstract knowledge of sound perception also makes direct questioning more difficult, since children at a younger age do not have the vocabulary or mental development to explain pitch, loudness, or localization. Such limitations lead to the inappropriate characterization of symptoms or delayed characterization at a clinical encounter [12]. Subjective reporting is a very critical part of tinnitus examination, but its reliability changes depending on different stages of development. Children of a younger age can be dependent on parenting interpretation, and it can add more doubt [8]. Differences in attention span, interest in the task, and fatigue in audiological tests are also complicating factors, especially where a prolonged cooperation is needed, as in audiological tests such as the tonal test or psychoacoustic matching [24].

**Figure 1:**
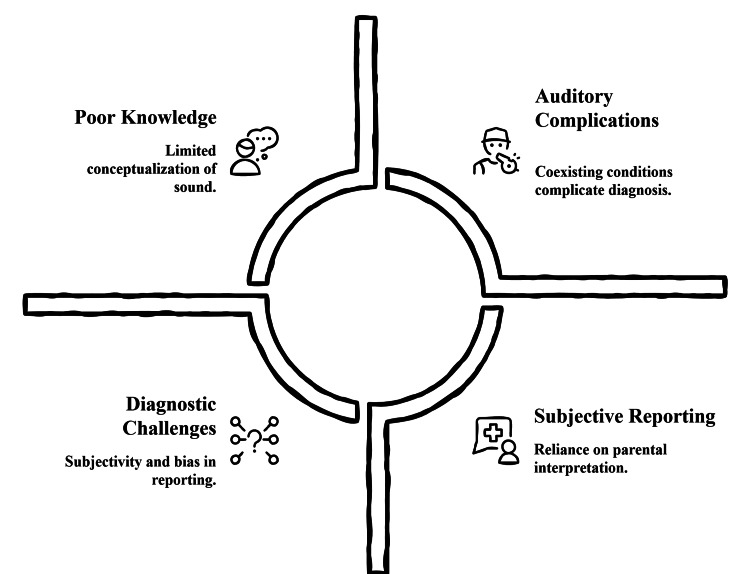
Diagnostic challenges of pediatric tinnitus Image created by the authors using Napkin AI (Los Altos, CA)

Comorbid auditory disorders add an added complexity in terms of diagnosis. The complaints related to tinnitus may be obscured or confused with secondary factors by conductive deficits related to otitis media, sensorineural complaints, vestibular disturbance, or noise exposure [4]. It can also be a developmental and neurocognitive disorder, such as attention-related disorders, autism spectrum disorders, or speech-language delays, that may interfere with behavioral reaction during the assessment, and it is not clear where or even how severe a perceived sound may be [25]. The objective diagnostic tools are still useful, but the interpretation of such diagnostic tools in children is delicate, owing to the fact that the children are still in the process of auditory maturation and also because of the variability of the anatomy [21]. Diagnostic refinement can be supported with the aid of otoacoustic emissions, auditory brainstem responses, and measures of middle-ear reflexes, but the results are not always consistent with the subjective perception. These issues underscore the importance of age-related approaches, empathetic communication methods, and holistic assessment models that are tailored in such a way that they can help in capturing the complex nature of tinnitus among children [25]. Figure 1 shows key clinical, developmental, and contextual barriers that hinder accurate diagnosis of tinnitus in children.

Screening and case-finding methods

Diagnosis of tinnitus among children is based on the systematic detection of signs of auditory disturbances in children or on subtle behavioral signs that indicate internal auditory perception [26]. Hearing screening programs implemented in schools can play a vital role in early detection because they are able to detect any threshold changes, any abnormalities of the middle ear, or any unexplained auditory complaints overall, which could be accompanied by tinnitus [22]. These programs often become an initial point of contact with children who have not yet received a formal audiological evaluation and provide systematic opportunities to identify abnormalities of anticipated auditory performance [18]. Regular check-ups in the pediatric practice can also play a significant role in case-finding because a clinician can note communication patterns, sleeping issues, or attention deficit, which are in line with the complaints of tinnitus [3]. Specialized interrogation in such interactions may reveal symptoms that otherwise go undetected because of poor vocabulary or lack of knowledge about the meaning of internal auditory phenomena. Observations of parents might be further used to inform screening by observing irritability, less engagement in quiet activities, or sound-avoidant behavior [27].

Developmentally relevant instruments are used by the audiologists in an attempt to narrow down the initial findings during school or clinic screening [21]. Middle ear fluid, subtle cochlear involvement, or reduced auditory sensitivity that may be associated with tinnitus can be identified with the help of tympanometry, screening otoacoustic emissions, or simplified tonal tests [16]. Children detected by these ways usually need a complete diagnostic process in order to define the characteristics of tinnitus, the functional effect, and the underlying factors. Combined screening methods in educational, pediatric, and audiological environments enhance the detection of cases early and encourage the prompt referral to the specialized examination [25]. Table 2 presents important screening procedures, areas of emphasis, results, and referral indicators applied in determining tinnitus in children.

**Table 2:** Screening strategies for early detection of pediatric tinnitus Compiled by the authors based on information synthesized from [8,22,25,27]. OAE, otoacoustic emissions

Screening Method	Target Population	Primary Focus	Typical Findings	Referral Trigger	References
School hearing screening	School-aged children	Threshold shifts and middle ear status	Mild loss, abnormal tympanogram	Persistent auditory complaints	[22]
Pediatric clinic visit	Infants to adolescents	Behavioral cues and developmental status	Irritability, sleep disturbance	Reported internal sound perception	[8]
OAE screening	All age groups	Cochlear outer hair cell function	Reduced or absent emissions	Suspected cochlear involvement	[25]
Tympanometry	Young children	Middle ear pressure and mobility	Fluid or negative pressure	Recurrent middle ear signs	[27]

Audiological diagnostic tools and behavioral assessments

Pediatric tinnitus audiological assessment is based on structured diagnostic tools aimed at assessing the integrity of the auditory pathway, as well as perceptual and response patterns of behavior at different developmental stages [28]. Pure-tone audiometry is still one of the main elements in children with the capacity to respond conditionally, giving threshold information at various frequencies that can indicate sensorineural or conductive involvement related to tinnitus [6]. The additional information provided by speech audiometry is the determination of the speech detection and word recognition skills that can provide insight into the functional level of listening in the real-life environment [11]. In younger ages, behavioral pure-tone methods of visual reinforcement audiometry and conditioned play audiometry allow the examiners to get accurate thresholds by developmentally adapted task formats. The methods help assess the hearing situation when the verbal reporting is restricted, and, in many cases, the methods are a key to match behavioral performance with potential tinnitus complaints [17].

Objective measures enhance the interpretation of the diagnosis because they assess auditory functions without the involvement of behavioral responses. Otoacoustic emissions can tell about the activity of cochlear outer hair cells, and thus it is possible to detect the initial cochlear disruption despite the normal behavioral threshold [15]. Auditory brainstem responses evaluate the existence of neural conduction along the auditory pathway in the brainstem cochlear nerve to the brainstem and help identify neural synchrony disturbances, retarded transmission, or postcochlear relevance, which may be present with tinnitus [23]. The concomitant application of behavioral and objective tests enhances the level of diagnostic accuracy because the two techniques provide distinct information to represent varying degrees of auditory processing [29]. Developmental stage, attention span, and task engagement are important elements that should be considered in interpretation, as differences in cooperation can affect reliability. Combined assessment in hearing aids in detecting changes in auditory tinnitus attributes, elucidating physiologic factors, and informing the choice of suitable audiologic intervention in the pediatric population [30].

Objective measurement techniques and emerging technologies

The objective assessment methods are useful in pediatric tinnitus since they give data about the auditory system functioning without self-report. The acoustic reflex test assesses the stapedius reflex pathway and provides information about the integrity of the middle ear and lower brain stems [31], and abnormal reflex patterns indicate changes in transmission of the neural transmission or increased auditory sensitivity linked with tinnitus [9]. Neural synchrony, response latency, and efficiency of the cochlea to the higher auditory centers are measured using electrophysiological techniques such as auditory brainstem responses and cortical auditory evoked potentials, and neural abnormalities are also detected that would otherwise be undetected using behavioral tests [32].

**Figure 2:**
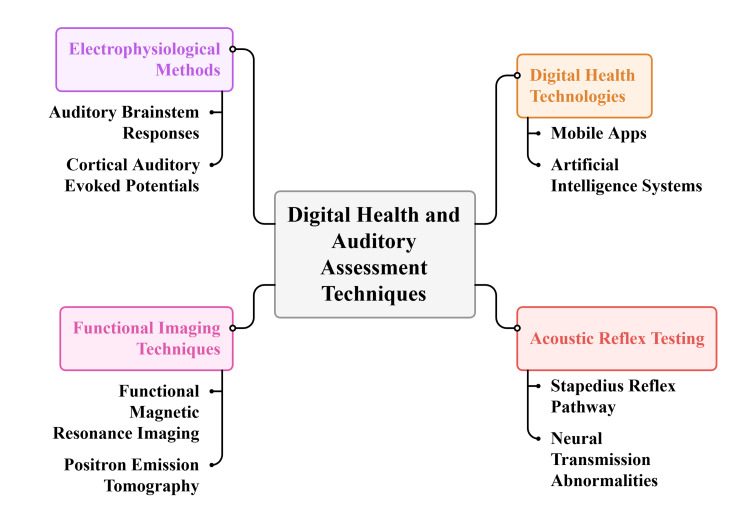
Objective assessment techniques for detecting pediatric tinnitus Image created by the authors using Napkin AI (Los Altos, CA)

Functional imaging also defines tinnitus perception patterns of the neural activity. Functional magnetic resonance imaging and positron emission tomography show increased activity in the auditory cortices, thalamic networks, and limbic structures through interaction between the auditory and emotional processing systems [14]. Digital health technologies enhance diagnostic power, and mobile apps allow tracking the symptoms and identifying patterns in real-time [27], and artificial intelligence-based systems facilitate automated signal processing and multimodal data integration [26]. The methods can supplement subjective evaluation by promoting individualized clinical decision-making, endorsing symptom profiling, and tracking treatment responses (habituation) in disease treatment [28,33]. Figure 2 shows important objective and technology-based interventions in the assessment of tinnitus in children.

Medical and otologic evaluation pathways

Pediatric tinnitus may be evaluated medically and otologically by systematically evaluating structures that can interfere with auditory functioning. Early otoscopic observation permits the view of the outer ear canal and tympanic membrane [34], whereby cerumen impaction, inflammation, perforation, or middle ear fluid are possible indications of conductive factors that can cause tinnitus sensation [30]. Tympanometry and acoustic reflex testing are also used in the identification of imbalance in the middle ear pressure, effusion, and ossicular restriction related to conductive involvement [26]. Clinical assessment is also done in relation to common associated conditions such as otitis media, dysfunction of the tympanic membrane, congenital abnormalities, external ear or middle ear infections, trauma, and ototoxic exposure [35]. Differentiation between acute and chronic patterns is supported by detailed history about the recent illness, head injury, noise exposure, or medication use [31]. Cranial nerve observation and cervical muscle and temporomandibular joint mobility would help in determining somatosensory factors that may control tinnitus intensity or localization [28].

Radiological justification is only undertaken in case of suspected structural aberration or retrocochlear involvement. Computed tomography is used to visualize middle ear abnormalities, mastoid abnormalities, ossicular abnormalities, or congenital abnormalities [36], whereas magnetic resonance imaging is used to give a detailed assessment of the cochlear nerve, internal auditory canal, and central auditory pathways to rule out lesions or demyelinating processes. The accurate interpretation and management planning are achieved with the help of the multidisciplinary collaboration of otolaryngologists, pediatricians, neurologists, and audiologists [37]. The summary of critical clinical assessment steps, diagnosis focus, common results, implications, and signs of additional testing of pediatric tinnitus is shown in Table 3.

**Table 3:** Components of medical and otologic evaluation in pediatric tinnitus Compiled by the authors based on information synthesized from [20,24,30,32,35]. CT, computed tomography; MRI, magnetic resonance imaging

Evaluation Step	Primary Focus	Typical Findings	Clinical Implication	Indication for Further Testing	References
Otoscopy	External canal and tympanic membrane	Cerumen impaction, fluid, infection, perforation	Identification of conductive factors	Persistent symptoms or abnormal appearance	[35]
Tympanometry	Middle ear pressure and mobility	Effusion, negative pressure	Confirmation of middle ear involvement	Recurrent middle ear dysfunction	[24]
Cranial nerve exam	Neural integrity	Facial weakness or sensory deficits	Indication of neurologic contribution	Suspicion of retrocochlear pathology	[30]
CT scan	Bone and middle ear structures	Congenital defects, ossicular issues	Structural clarification	Trauma or suspected malformation	[32]
MRI	Nerve and central auditory pathways	Neural lesions or inflammation	Central pathway evaluation	Unexplained or persistent tinnitus	[20]

Psychological and neurocognitive assessment

The neurocognitive/psychological assessment is significant in pediatric tinnitus evaluation since emotions and cognitions have roles in the perceptions of the symptoms and functional effects [38]. Anxiety, stress, irritability, and sleep disturbance screening can determine children with high tinnitus awareness or insufficient coping ability [25], even with those having elevated sensory sensitivity [31]. Tinnitus also has the potential to damage attention, working memory, and executive functioning, which leads to academic and task-related problems, with standardized testing able to identify a deficiency in attention, slower processing speed, and task persistence [29,33]. Due to the continuous emotional growth, which occurs throughout the childhood period, distress caused by tinnitus potentially becomes more severe due to the lack of knowledge of the inner sensations, leading to growing worry and avoidance behaviors [39]. Associations between mood, coping style, and tinnitus burden, as well as the integration of psychological and neurocognitive results make clinical interviews and age-relevant psychological scales explain why developmentally informed management reduces distress and enhances adaptive coping [35,40].

Evidence-based treatment strategies

Pediatric tinnitus therapeutic intervention is intended to stabilize the auditory system, alleviate suffering, and enhance adaptive coping. Sound-based interventions are still the focus as controlled sound enrichment decreases the difference between silence and inner sound audition [41] in favor of habituation by low-level background sound, therapeutic noise programs, or facilitated enrichment strategies [35]. Hearing aids can be of help in children with hearing impairment who have hearing difficulties because these devices enhance the audibility of speech and reduce the level of tinnitus due to increased external stimulation [19]. Tinnitus retraining therapy is a type of treatment that involves exposure to sound and counselling to lessen attention given to tinnitus and induce neural adaptation at a lower limbic involvement [34]. Cognitive-behavioral strategies are aimed at negative emotional and cognitive reactions, which enhance anxiety, disruption in sleep, coping capacity, and quality of life [33].

Mindfulness-based interventions and relaxation training can also help decrease sensory tension and enhance autonomic regulation, especially among older children and adolescents who can participate in guided practice [42]. The application of pharmacological interventions is cautious because of developmental sensitivity and unstable reactions to drugs [26], and even though drugs could help cope with sleep disturbance, anxiety, or underlying otologic pathophysiology, the evidence on the direct anti-tinnitus effect is still scarce [37]. The choice of treatment is determined by the severity of symptoms, level of development, comorbidities, and tolerance, and usually best results are obtained after a multidisciplinary audiological, psychological, and medical treatment approach [43].

Multidisciplinary management and long-term care models

Pediatric tinnitus as a multidisciplinary problem demands the concerted efforts of clinical disciplines due to the fact that the effects of tinnitus can include auditory, psychological, and developmental sectors [44]. Otolaryngologists detect any structural or inflammatory causes and inform medical or surgical management where necessary [32]. Audiologists perform hearing assessment, hearing surveillance, and individual-profile sound-based interventions or amplification. Psychologists deal with emotional distress, coping patterns, sleep disturbance, and attentional problems by employing systematic approaches to decrease distress and enhance self-regulation [40]. Pediatricians endorse overall health, as well as developmental and possible etiological state tracking such as recurrent infections or persistent inflammation [38]. Neurologists might be engaged in cases of tinnitus associated with central auditory processing, neural irritability, or syndromes related to headache. One of the roles played by speech-language pathologists is in determining communication and language variables that can affect the reporting of symptoms and daily functioning [41].

Long-term management is based on family-centered care where the caregiver provides advice on symptom interpretation, effective environment management, and support for behavioral change, enhancing home-based coping [37]. Teacher training and school personnel encourage learning environment modifications, decrease the load on concentration, and facilitate academic activity [42]. The models of long-term care are based on periodic follow-up due to the possible fluctuations in tinnitus presentation and functional influence depending on auditory, neural, and psychosocial developments. Continuous monitoring enables the management strategies to be reviewed in time and emerging concerns to be identified promptly [43]. Coherent medical, audiological, psychological, and educational services offer flexible support of developmental stages to enhance functional results during childhood and adolescence [45].

Limitations and future recommendations

Evidence on pediatric tinnitus is limited due to the lack of longitudinal data, small groups, the absence of consistent diagnostic approaches, and differences in the methods of assessing it in clinical settings. The available evidence is usually due to heterogeneous populations, which minimizes clarity when it comes to developmental differences and limits the generalizability. Instruments of objective assessment that are appropriate in children are not well standardized, and emotional or cognitive factors are not adequately assessed because of the limited number of validated instruments in pediatrics, reducing the accuracy of clinical interpretation.

Further developments would be enhanced by particle interactions that use common diagnostic guidelines and more age-specific groups with regular longitudinal follow-up to elucidate the development of the symptoms and sensitivity to treatment. Wider justification of objective instruments at different levels of development can increase diagnostic trustworthiness. Greater research on digital health platforms, symptom-tracking apps, and analysis using artificial intelligence can reinforce the early detection and individual care. A combination of psychological, educational, and neurodevelopmental approaches to long-term management models can further narrow down on avenues to effective and age-based tinnitus management.

## Conclusions

Pediatric tinnitus is a complex auditory disorder, which is developmentally, physiologically, and psychologically conditioned and needs coordinated clinical management. It is important to identify it earlier, as symptom expression might be revealed in a subtle way and influence communication, learning and emotional control, and functioning in general. Combined strategies of behavioral measurements, objective auditory devices, medical assessment, and psychological assessment increase the accuracy of the diagnosis, allowing the identification of underlying contributors on peripheral and central pathways. Multimodal approaches, including sound-based interventions, amplification where required, structured counselling, cognitive interventions, and supportive environmental adaptations based on each developmental stage, are used to enhance treatment outcomes. The success of multidisciplinary care (otolaryngology, audiology, psychology, pediatrics, neurology, and educational services) is the basis of successful management and long-term follow-up, as symptom characterization may change with age. The development of digital health technologies, objective measurements, and symptom-monitoring devices provide promising opportunities to improve diagnostic accuracy and personalized care. Further work on the standardized protocols and the model of long-term care is critical to the enhancement of the outcomes and the promotion of the specialized auditory and neurocognitive needs of children with tinnitus.
